# Undiagnostic Heart Failure With Preserved Ejection Fraction in Atrial Fibrillation Patients Undergoing Catheter Ablation

**DOI:** 10.1002/joa3.70258

**Published:** 2025-12-26

**Authors:** Masanaru Sawada, Ryuta Watanabe, Koichi Nagashima, Yuji Saito, Yuji Wakamatsu, Naoto Otsuka, Koichiro Hori, Shu Hirata, Moyuru Hirata, Yasuo Okumura

**Affiliations:** ^1^ Division of Cardiology, Department of Medicine Nihon University School of Medicine Tokyo Japan

**Keywords:** atrial fibrillation, catheter ablation, diastolic dysfunction, heart failure with preserved ejection fraction, HFA‐PEFF score

## Abstract

**Background:**

Atrial fibrillation (AF) and heart failure with preserved ejection fraction (HFpEF) often coexist, but the prevalence of HFpEF among AF patients undergoing catheter ablation (CA) remains unclear.

**Methods:**

We studied 127 AF patients with preserved ejection fraction (≥ 50%) undergoing initial CA. The Heart Failure Association Pre‐test assessment, Echocardiography and natriuretic peptides, Functional testing, and Final etiology (HFA‐PEFF) score was assessed 2 weeks before and 1 year after CA. Patients were grouped as low (0–1), intermediate (2–4), or high (5–6). The primary endpoint was AF freedom at 1 and 2 years; secondary endpoints were changes in HFA‐PEFF score; tertiary endpoint was predictors of score improvement.

**Results:**

Of 127 patients, 30 (23.6%) had HFpEF (score ≥ 5), 76 (59.8%) suspected (2–4), and 21 (16.5%) unlikely (≤ 1). Median follow‐up 24.4[ 12.5–29.5] months. AF freedom at 1 year was high (86.4%, 91.7%, 89.7%; *p* = 0.66). Higher baseline score correlated with older age, female sex, hypertension, larger left atrial volume index (LAVI), elevated average *E*/*e*′, and increased left ventricular mass index. We divided patients into two groups: those with ≥ 1‐point score improvement after CA (*n* = 89, 70.1%) and those whose score remained unchanged or worsened (*n* = 38, 29.9%). Improvement was associated with older age, larger LAVI, higher average *E*/*e*′, and elevated N‐terminal pro‐B‐type natriuretic peptide. Multivariate analysis identified septal e' and LAVI as predictors. No patients had a score ≥ 5 at 1 year.

**Conclusions:**

HFpEF or suspected HFpEF was common in AF CA candidates but not linked to recurrence. CA remarkably improved HFpEF features, suggesting reversibility.

## Graphical abstract

Among 127 atrial fibrillation (AF) patients with preserved ejection fraction, 23.6% met HFpEF criteria by the HFA‐PEFF score before catheter ablation (CA), but none at 1 year. CA improved HFA‐PEFF scores independent of recurrence, suggesting that AF‐related HFpEF features are substantially reversible and highlighting the dual role of CA in rhythm control and modification of HFpEF‐related abnormalities.

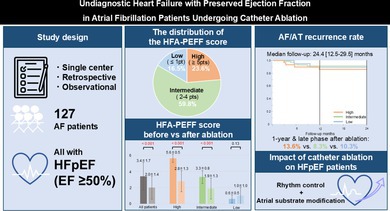

**Abbreviations**
ACE‐I: angiotensin‐converting enzyme inhibitor
AF: atrial fibrillation
ARB: angiotensin II receptor blocker
ARNI: angiotensin receptor–neprilysin inhibitor
AT: atrial tachycardia
ATP: adenosine triphosphate
BMI: body mass index
CA: catheter ablation
CF: contact force
CTI: cavotricuspid isthmus
DCM: dilated cardiomyopathy
HFA‐PEFF score: Heart Failure Association–Pre‐test assessment, Echocardiography and natriuretic peptides, Functional testing, and Final etiology score
HFpEF: heart failure with preserved ejection fraction
LAVI: left atrial volume index
LVEF: left ventricular ejection fraction
LVMI: left ventricular mass index
MRA: mineralocorticoid receptor antagonist
NT‐proBNP: N‐terminal pro‐B‐type natriuretic peptide
OMI: old myocardial infarction
PV: pulmonary vein
PVI: pulmonary vein isolation
RFCA: radiofrequency catheter ablation
RWT: relative wall thickness
SGLT2: sodium‐dependent glucose co‐transporter 2
SR: sinus rhythm
SVC: superior vena cava
TIA: transient ischemic attack
TIC: tachycardia‐induced cardiomyopathy
TR: tricuspid regurgitation

## 1 Introduction

Atrial fibrillation (AF) and heart failure with preserved ejection fraction (HFpEF) are two prevalent cardiovascular conditions that frequently coexist, especially in the aging population [1, 2]. The coexistence of these two conditions significantly worsens the clinical prognosis and complicates management strategies [3, 4, 5]. Catheter ablation (CA) has become a cornerstone rhythm‐control therapy for AF, and beyond arrhythmia suppression, recent evidence suggests that CA may confer beneficial effects on cardiac structure, function, and symptoms in patients with AF and heart failure [6, 7, 8]. These observations have increased clinical interest in understanding how HFpEF behaves in the context of AF and rhythm control therapy.

The HFA‐PEFF (Heart Failure Association‐Pre‐test assessment, Echocardiography and natriuretic peptides, Functional testing, and Final etiology) score has recently been established as a comprehensive diagnostic framework for HFpEF [9]. However, despite its growing adoption, important evidence gaps remain. First, the true prevalence of HFpEF and suspected HFpEF among patients referred for AF ablation has not been fully characterized in real‐world settings. Second, whether baseline HFpEF severity influences rhythm outcomes after CA is unclear. Third, it remains unknown to what extent CA modifies HFpEF‐related structural and biochemical abnormalities and which clinical features predict improvement.

To address these gaps, this study aimed to: (1) determine the prevalence of HFpEF and suspected HFpEF in AF ablation candidates using the HFA‐PEFF scoring system; (2) compare AF/atrial tachycardia (AT) recurrence rates at 1 and 2 years after CA according to baseline HFA‐PEFF score categories; and (3) evaluate changes in the HFA‐PEFF score and its individual components one year after ablation and to identify baseline predictors associated with score improvement.

## 2 Methods

### 2.1 Study Design

**FIGURE 1:**
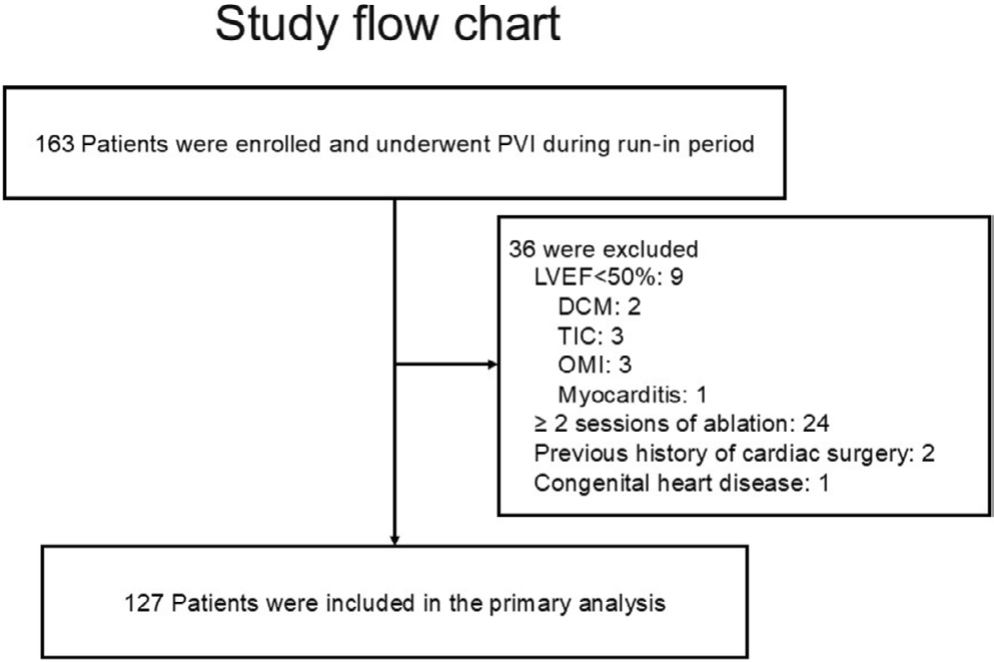
Flow diagram of patient enrollment and exclusion. Of 163 patients enrolled, 36 were excluded due to reduced LVEF (< 50%), history of multiple ablations, prior cardiac surgery, or congenital heart disease. DCM, dilated cardiomyopathy; LVEF, left ventricular ejection fraction; OMI, old myocardial infarction; TIC, tachycardia‐induced cardiomyopathy.

This was a retrospective analysis of prospectively and uniformly managed patients undergoing CA under a standardized protocol at a single center. The study participants included 163 consecutive patients who had undergone CA of paroxysmal AF (PAF; defined as AF returning to sinus rhythm (SR) within 7 days), persistent AF (PerAF; defined as AF lasting ≥ 7 days) at Nihon University Itabashi Hospital between February and October 2022. Patients with preserved ejection fraction, regardless of heart failure symptoms, who underwent initial AF ablation were included, and those who underwent CA were given transthoracic echocardiography and blood tests 2 weeks before and 1 year after CA. Exclusion criteria were (1) patients with reduced left ventricular ejection fraction (LVEF < 50%) due to dilated cardiomyopathy (DCM), tachycardia‐induced cardiomyopathy (TIC), old myocardial infarction (OMI), or myocarditis, (2) patients who had undergone ≥ 2 sessions of CA, (3) patients with previous history of cardiac surgery and (4) a patient with congenital heart disease. Of a total of 163 patients, we excluded 9 with LVEF < 50%, 24 who had ≥ 2 sessions of CA, 2 who had a previous history of cardiac surgery, and 1 who had congenital heart disease. As a result, 127 patients were enrolled in this study for the final analysis (Figure 1). This study conformed to the Declaration of Helsinki and the Ethical Guidelines for Clinical Studies issued by the Ministry of Health, Labour, and Welfare, Japan. All participants provided written informed consent and could withdraw their consent at any time. This study protocol was approved by the Institutional Review Board (IRB) of Nihon University Itabashi Hospital, Clinical Research Judging Committee.

### 2.2 Clinical Data Collection

The patient information including the age, male sex, height, body weight, presence or absence of AF‐related symptoms (defined as at least one of the following: palpitations, dizziness, rapid heartbeat, dyspnea, or syncope, excluding fatigue) and CHADS_2_ score components (history of heart failure, hypertension, diabetes, or stroke/transient ischemic attack) was collected prior to CA. Symptom status was assessed during outpatient clinic visits. Laboratory data included the N‐terminal pro‐B‐type natriuretic peptide (NT‐proBNP). The echocardiographic parameters included the septal *e*′, lateral *e*′, average *E*/*e*′, TR velocity, left atrial volume index (LAVI), left ventricular mass index (LVMI), relative wall thickness (RWT), and LV wall thickness. Blood tests and transthoracic echocardiography were performed 2 weeks before and 1 year after CA.

### 2.3 Ablation Methods

For all patients, antiarrhythmic drugs were discontinued for at least 5 half‐lives prior to the CA procedure, and oral anticoagulants were generally discontinued on the day of CA. Conscious sedation was achieved with dexmedetomidine, propofol, and fentanyl. Vascular access was obtained, a single transseptal puncture guided by intra‐cardiac ultrasound was performed, and intravenous heparin was administered to maintain an activated clotting time of > 300 s. Three‐dimensional maps of the left atrium and 4 pulmonary veins (PVs) were created with CARTO 3 (Biosense Webster) or the NavX system (Abbott Laboratories, Abbott Park, IL). An extensive encircling pulmonary vein isolation (PVI) was guided by a circular mapping catheter or multiple‐electrode catheter and a 3D mapping system. The ablation catheter was an irrigated‐tip contact force (CF) sensing catheter (SmartTouch SF [Biosense Webster Inc.] or TactiCath [St. Jude Medical, St. Paul, MN]) with a target CF of 10–15 g, and the ablation settings were based on an ablation index of 450 or lesion index of 5.5 for anterior sites and an ablation index of 350–400 or lesion size index of 4.5–5.0 for posterior sites with a power of 35 W. Regarding the balloon ablation technologies, an Arctic Front Advance cryoballoon (Medtronic, Minneapolis, MN, USA) was used as described previously [10, 11, 12]. Any touch‐up ablation required for dormant conduction or residual PV potentials was performed with a standard irrigated‐tip catheter. In all patients without contraindications to adenosine triphosphate (ATP), intravenous ATP was administered after PVI to identify any dormant PV conduction. If acute PV reconnections or dormant PV conduction was evident, touch‐up ablation was performed. In patients with long‐standing AF or those with advanced left atrial remodeling, box isolation was performed at the operator's discretion. Cavotricuspid isthmus ablation was performed in patients with clinically documented typical atrial flutter or when it was inducible during the procedure, and superior vena cava isolation was performed only when spontaneous firing from the SVC was observed intra‐procedurally.

### 2.4 Post‐Ablation Follow‐Up

On the day after the CA procedure, all antiarrhythmic drugs previously prescribed were resumed at the individual operator's discretion. Patients were followed at our hospital outpatient clinics with physical examinations and 12‐lead ECGs at 1, 3, 6, 12 months, and annually thereafter. Twenty‐four‐hour Holter monitoring was performed at 3–6, 12 months, and annually thereafter. They were simultaneously followed up at their respective outpatient clinics every 1–3 months. Any symptomatic or documented atrial arrhythmias of ≥ 30 s after a 3‐month blanking period were taken as a recurrence of the AF/AT.

### 2.5 Study Endpoint

The primary endpoint was the AF freedom rate at 1 and 2 years following CA. The secondary endpoints were the changes in HFA‐PEF score and various parameters included in the score before and 1 year after CA. The tertiary endpoint was the factors that contribute to the improvement of the HFA‐PEF score at 1 year by CA for AF.

### 2.6 Statistical Analysis

Categorical variables are presented as the number and percentage of patients. Continuous variables are presented as mean ± standard deviation or median with interquartile range, as appropriate. Between‐group differences among the three groups stratified by HFA‐PEFF score (0–1, 2–4, and ≥ 5) were analyzed using one‐way analysis of variance (ANOVA) for normally distributed variables or the Kruskal–Wallis test for non‐normally distributed variables. Categorical variables were compared using the chi‐square (*χ* ^2^) test or Fisher's exact test, as appropriate; the latter was applied when the expected frequency in any cell was less than 5. The cumulative event rate was estimated by the Kaplan–Meier method, and the differences were analyzed by a log‐rank test. Univariable and multivariable logistic regression analyses were performed to identify determinants of HFA‐PEFF score improvement. Variables were selected based on their clinical importance and/or statistical significance in univariable testing. Because average *E*/*e*′ demonstrated collinearity with both LAVI and NT‐proBNP, septal *e*′ was selected as the representative diastolic parameter in the final model. The multivariable model included age, septal *e*′, LAVI, and log‐transformed NT‐proBNP. NT‐proBNP values were log‐transformed prior to analysis to account for their skewed distribution. The association between rhythm changes from baseline to 1 year and HFA‐PEFF score improvement was assessed using the Fisher–Freeman–Halton test. In addition, the Mann–Whitney *U* test was used to compare the degree of HFA‐PEFF score improvement between patients who converted from AF to SR and those who maintained SR throughout. All statistical analyses were performed with JMP 13.2.1 software (SAS Institute, Cary, NC), and a *p* < 0.05 was considered significant.

## 3 Results

### 3.1 Distribution of HFA‐PEFF Scores and Baseline Characteristics

**FIGURE 2:**
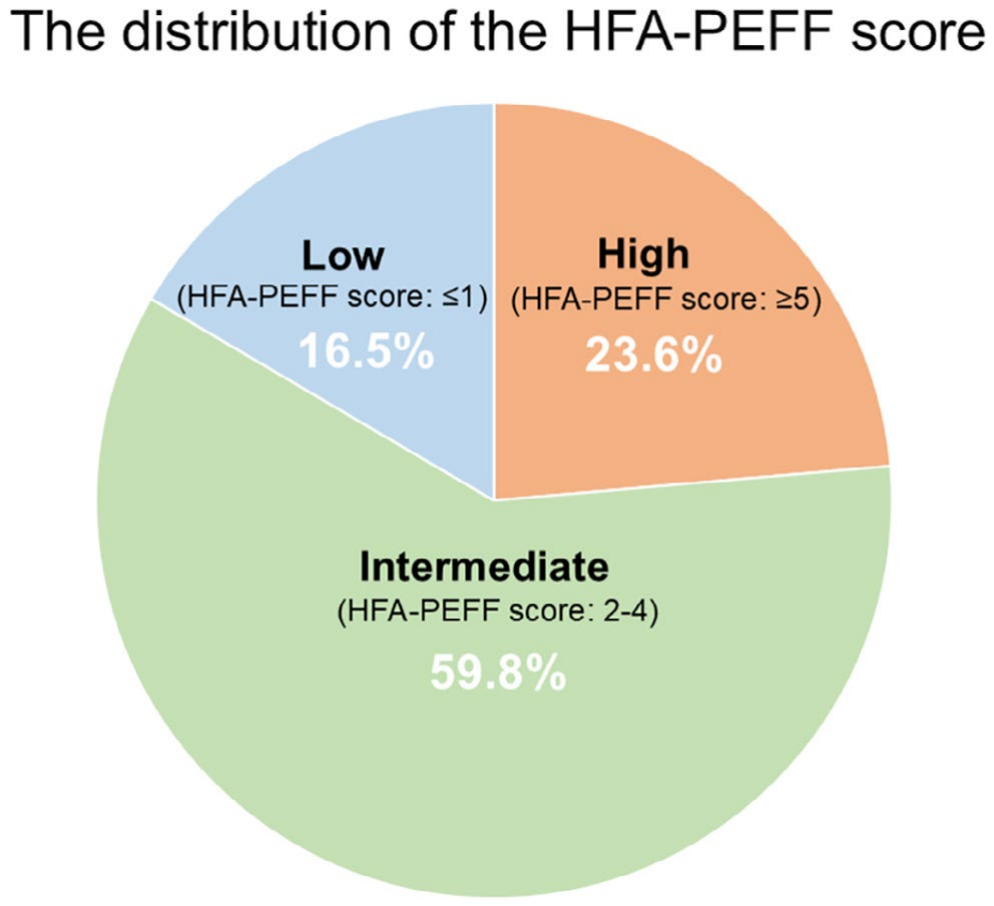
Pie chart illustrating the distribution of HFA‐PEFF scores prior to CA. Among the 127 patients analyzed, 23.6% had scores ≥ 5 (HFpEF), 59.8% had intermediate scores (2–4), and 16.5% had low scores (≤ 1). CA, catheter ablation; HFpEF, heart failure with preserved ejection fraction.

Figure 2 shows the distribution of baseline HFA‐PEFF scores. Before CA, 30 patients (23.6%) fulfilled HFpEF criteria (score ≥ 5), 76 (59.8%) had intermediate scores (2–4), and 21 (16.5%) had low scores (0–1). Baseline characteristics across the score groups are summarized in Table 1. Compared with the intermediate and low‐score groups, patients with HFA‐PEFF ≥ 5 were older, more frequently female, had higher CHA_2_DS_2_‐VASc scores, and showed significantly worse echocardiographic and biomarker parameters (lower septal e', higher average E/e', larger LAVI, higher LVMI, and higher NT‐proBNP). Use of renin–angiotensin system inhibitors and SGLT2 inhibitors was more frequent in the HFpEF group.

**TABLE 1:** Baseline patient clinical, laboratory data, and echocardiographic characteristics per study group.

	Total	HFA‐PEFF score: ≥ 5	HFA‐PEFF score: 2–4	HFA‐PEFF score: ≤ 1	*p*
		*N* = 30	*N* = 76	*N* = 21	
**Clinical characteristic**					
Age (years)	65.5 ± 11.5	71.4 ± 8.3	66.2 ± 9.9	54.2 ± 13.5	< 0.001
Sex (male)	92 (72.4%)	17 (56.7%)	57 (75.0%)	18 (85.7%)	0.05
Height (cm)	165.3 ± 9.0	161.0 ± 8.8	165.7 ± 8.8	170.2 ± 7.2	0.001
Body weight (kg)	65.2 ± 11.9	61.6 ± 12.0	65.1 ± 10.7	71.0 ± 13.9	0.02
BMI (kg/m^2^)	23.8 ± 3.3	23.6 ± 3.4	23.6 ± 3.1	24.4 ± 4.1	0.60
Symptomatic	88 (69.3%)	23 (76.7%)	54 (71.1%)	11 (52.4%)	0.16
**AF type**					
Paroxysmal AF	68 (53.5%)	19 (63.3%)	40 (52.6%)	9 (42.9%)	0.34
Persistent AF	59 (46.5%)	11 (36.7%)	36 (47.4%)	12 (57.1%)	0.34
CHADS_2_ score	1.2 ± 1.0	1.7 ± 1.2	1.1 ± 0.9	0.6 ± 0.7	< 0.001
CHA_2_DS_2_‐VASc score	2.1 ± 1.4	3.0 ± 1.5	2.0 ± 1.3	1.1 ± 0.8	< 0.001
**Comorbidities**					
Chronic heart failure	22 (17.3%)	9 (30.0%)	11 (14.5%)	2 (9.5%)	0.10
Hypertension	69 (54.3%)	20 (66.7%)	41 (54.0%)	8 (38.1%)	0.13
Diabetes mellitus	15 (11.8%)	3 (10.0%)	11 (14.5%)	1 (4.8%)	0.45
Stroke/TIA	12 (9.4%)	5 (16.7%)	7 (9.2%)	0 (0.0%)	0.13
**Antiarrhythmic drug use at baseline**					
Class I	29 (22.8%)	3 (10.0%)	18 (23.7%)	8 (38.1%)	0.06
Class II	41 (32.3%)	15 (50.0%)	19 (25.0%)	7 (33.3%)	0.05
Class III	1 (0.8%)	0 (0.0%)	1 (1.3%)	0 (0.0%)	0.68
Class IV	32 (25.2%)	11 (36.7%)	19 (25.0%)	2 (9.5%)	0.09
**Medications at baseline**					
SGLT2i	11 (8.7%)	6 (20.0%)	5 (6.6%)	0 (0.0%)	0.03
MRA	7 (5.5%)	3 (10.0%)	3 (4.0%)	1 (4.8%)	0.46
ACE‐I or ARB or ARNI	32 (25.2%)	12 (40.0%)	18 (23.7%)	2 (9.5%)	0.04
**HFA‐PEFF score**	3.4 ± 1.7	5.7 ± 0.5	3.3 ± 0.8	0.6 ± 0.5	< 0.001
Septal *e*′ (cm/s)	8.2 ± 2.8	6.4 ± 1.5	8.1 ± 2.4	11.1 ± 3.2	< 0.001
Lateral *e*′ (cm/s)	11.1 ± 3.5	8.7 ± 2.7	11.3 ± 3.4	13.8 ± 2.6	< 0.001
Average *E*/*e*′	8.3 ± 3.4	11.6 ± 4.3	7.8 ± 2.3	5.6 ± 1.5	< 0.001
TR velocity (m/s)	2.2 ± 0.3	2.3 ± 0.3	2.2 ± 0.3	2.1 ± 0.3	0.16
LAVI (mL/m^2^)	39.0 ± 15.3	49.0 ± 13.7	38.0 ± 14.9	26.0 ± 5.0	< 0.001
LVMI (g/m^2^)	90.9 ± 25.9	98.2 ± 31.0	91.2 ± 26.0	79.8 ± 10.7	0.04
RWT	0.4 ± 0.1	0.4 ± 0.1	0.4 ± 0.1	0.4 ± 0.1	0.23
LV wall thickness (mm)	9.7 ± 1.4	9.9 ± 1.8	9.6 ± 1.2	9.1 ± 1.1	0.12
EF (%)	66.9 ± 6.8	66.2 ± 8.7	67.1 ± 6.2	67.1 ± 6.4	0.81
NT‐proBNP (pg/mL)	524 [83–666]	974 [239–1990]	174 [77–615]	63 [38–179]	< 0.001
**Procedural characteristics**					
Ablation modality					
RFCA	107 (84.3%)	25 (83.3%)	64 (84.2%)	18 (85.7%)	0.97
Cryoballoon Ablation	20 (15.7%)	5 (16.7%)	12 (15.8%)	3 (14.3%)	0.97
LA posterior wall isolation	14 (11.0%)	4 (13.3%)	9 (11.8%)	1 (4.8%)	0.59
SVC isolation	13 (10.2%)	4 (13.3%)	5 (6.6%)	4 (19.1%)	0.20
Mitral isthmus ablation	1 (0.8%)	0 (0.0%)	1 (1.3%)	0 (0.0%)	0.71
CTI ablation	20 (15.7%)	5 (16.7%)	11 (14.5%)	4 (19.1%)	0.87

*Note:* Mean ± SD or median (25th, 75th percentile) values or number (%) of patients are shown.

Abbreviations: ACE‐I, angiotensin‐converting enzyme inhibitor; ARB, angiotensin II receptor blocker; ARNI, angiotensin receptor–neprilysin inhibitor; BMI, body mass index; CTI, cavotricuspid isthmus; EF, ejection fraction; HFA‐PEFF score, The Heart Failure Association Pre‐test assessment, Echocardiography and natriuretic peptides, Functional testing, and Final etiology score; LAVI, left atrial volume index; LVMI, left ventricular mass index; MRA, mineralocorticoid receptor antagonist; NT‐proBNP, *N*‐terminal pro‐B‐type natriuretic peptide; RFCA, radiofrequency catheter ablation; RWT, relative wall thickness; SGLT2, sodium‐dependent glucose co‐transporter 2; SVC, superior vena cava; TIA, transient ischemic attack; TR, tricuspid regurgitation.

### 3.2 AF/AT Recurrence Rate Between Three Groups

**FIGURE 3:**
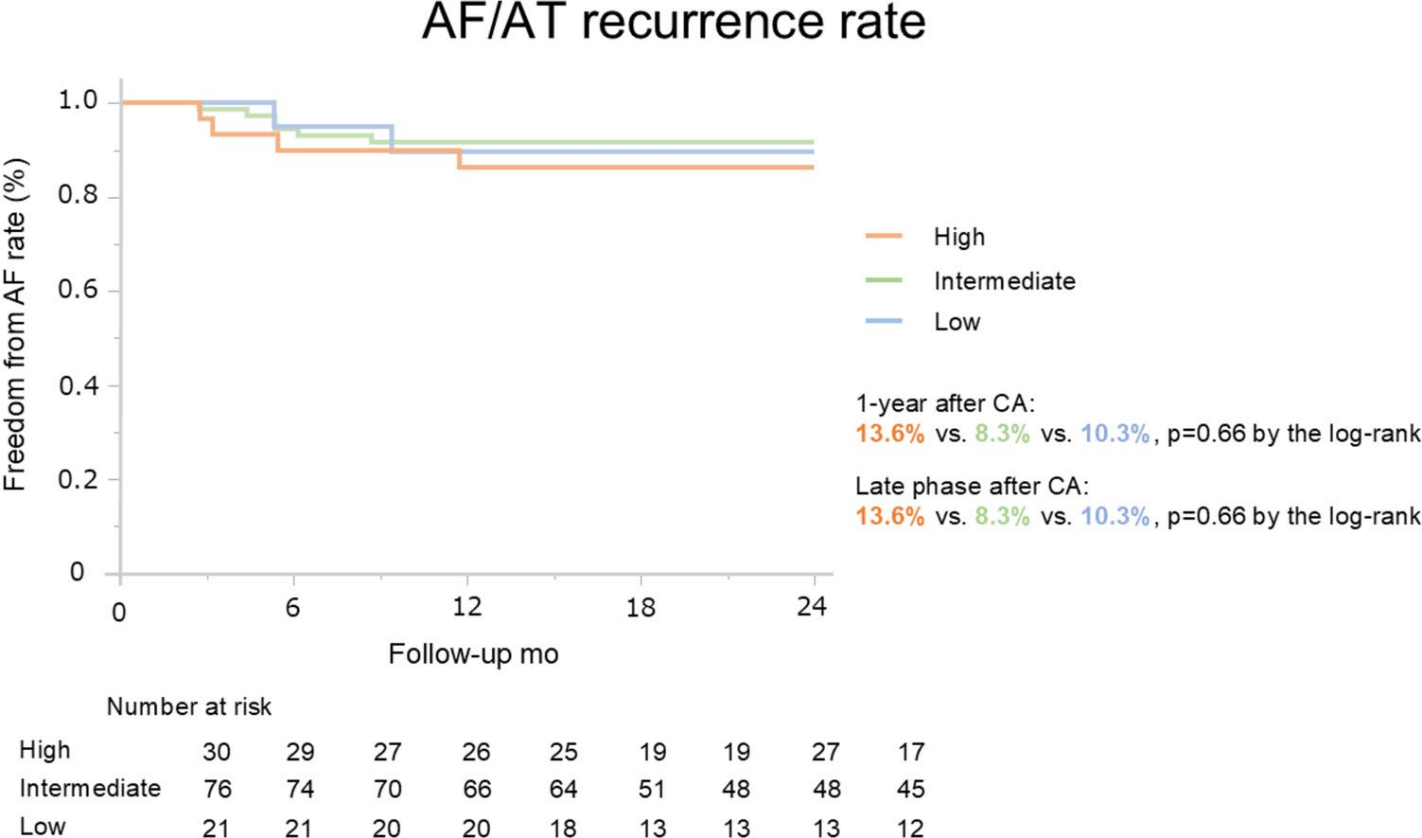
Freedom from AF/AT recurrence in patients stratified by baseline HFA‐PEFF score. No significant differences in arrhythmia‐free survival were observed at 12 months post‐ablation among the high (≥ 5), intermediate (2–4), and low (≤ 1) score groups (log‐rank *p* = 0.66). This trend remained unchanged at a median follow‐up of 24.4 months (log‐rank *p* = 0.66). AF, atrial fibrillation; AT, atrial tachycardia.

Figure 3 shows the Kaplan–Meier curves for freedom from AF/AT recurrence. Acute procedural success was achieved in all patients. At 1 year, freedom from recurrence was 86.4% in the HFA‐PEFF ≥ 5 group, 91.7% in the intermediate group, and 89.7% in the low‐score group (log‐rank *p* = 0.66). At 2 years (median follow‐up 24.4 months), recurrence‐free survival remained comparable among groups (log‐rank *p* = 0.66).

### 3.3 Post‐Ablation Changes in HFA‐PEFF Scores and Parameters

**FIGURE 4:**
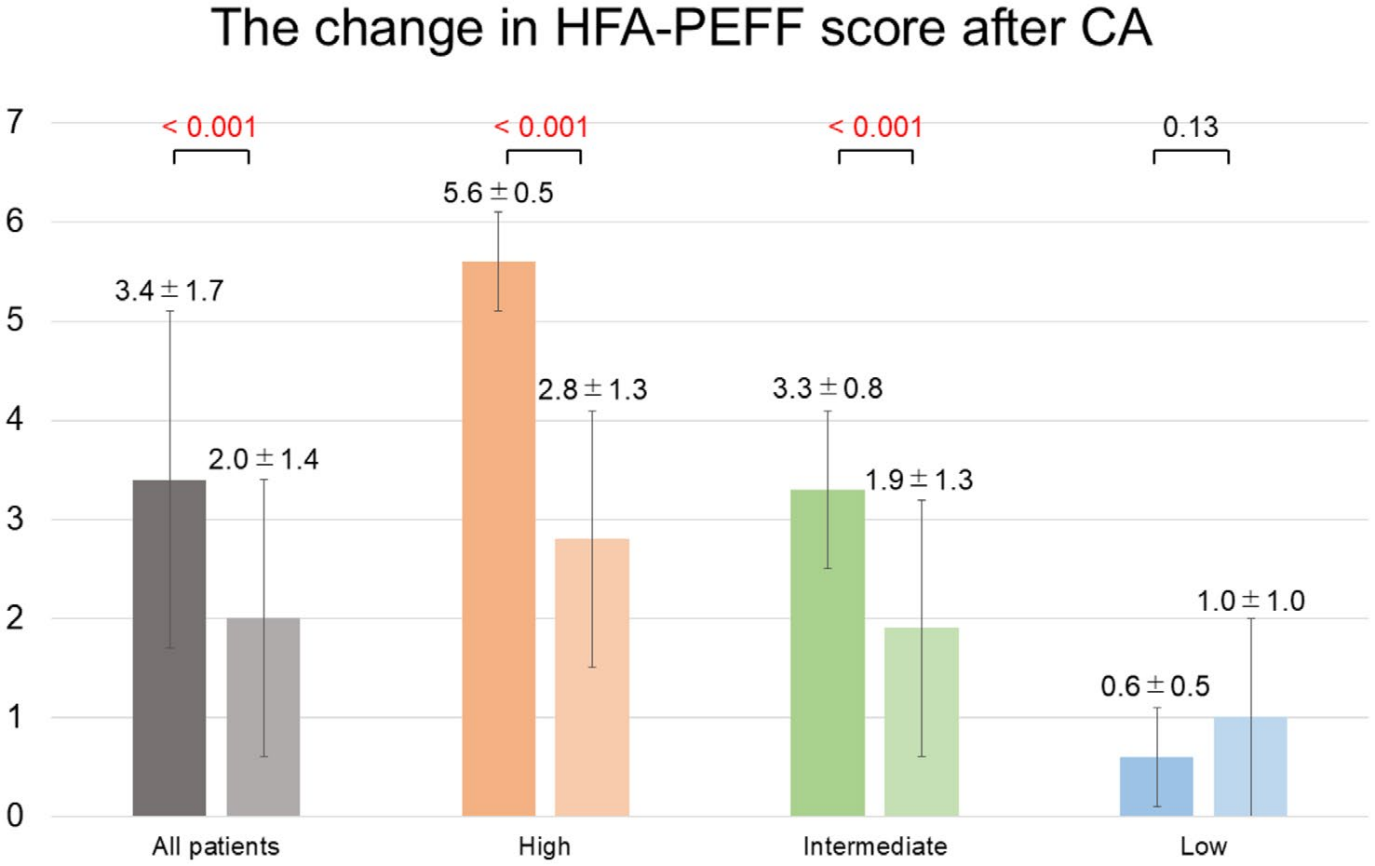
Longitudinal changes in HFA‐PEFF scores before and one year after CA across the three groups. Significant reductions in scores were observed in the high and intermediate groups (*p* < 0.001 for both), while the low‐score group showed no significant change (*p* = 0.13). CA, catheter ablation.

Figure 4 and Table 2 summarize changes after CA. HFA‐PEFF scores significantly decreased in the ≥ 5 and 2–4 groups (≥ 5: 5.6 ± 0.5 to 2.8 ± 1.3; 2–4: 3.3 ± 0.8 to 1.9 ± 1.3; both *p* < 0.001), while no significant change occurred in the ≤ 1 group (0.6 ± 0.5 to 1.0 ± 1.0; *p* = 0.13). Overall, 89 patients (70.1%) showed ≥ 1‐point score improvement. Importantly, none of the 30 patients with HFA‐PEFF ≥ 5 at baseline continued to meet diagnostic criteria at 1 year.

**TABLE 2:** The changes in the various parameters before and 1‐year after ablation per study group.

	HFA‐PEFF score: ≥ 5			HFA‐PEFF score: 2–4			HFA‐PEFF score: ≤ 1		
	*N* = 30			*N* = 76			*N* = 21		
	Pre	Post	*p*	Pre	Post	*p*	Pre	Post	*p*
HFA‐PEFF score	5.6 ± 0.5	2.8 ± 1.3	< 0.001	3.3 ± 0.8	1.9 ± 1.3	< 0.001	0.6 ± 0.5	1.0 ± 1.0	0.13
Septal *e*′ (cm/s)	6.4 ± 1.5	6.1 ± 2.3	0.39	8.1 ± 2.4	7.3 ± 1.8	0.007	11.1 ± 3.1	9.8 ± 2.7	0.08
Lateral *e*′ (cm/s)	8.7 ± 2.7	8.1 ± 2.7	0.14	11.3 ± 3.4	9.8 ± 2.2	< 0.001	13.8 ± 2.6	11.7 ± 2.3	< 0.001
*E*/*e*′	11.6 ± 4.3	11.0 ± 5.7	0.38	7.8 ± 2.3	7.7 ± 2.2	0.82	5.6 ± 1.5	6.2 ± 2.0	0.11
LAVI (mL/m^2^)	49.0 ± 13.7	39.3 ± 15.1	< 0.001	38.0 ± 14.9	30.4 ± 9.6	< 0.001	26.0 ± 5.0	24.0 ± 6.3	0.42
LVMI (g/m^2^)	98.2 ± 30.9	102.1 ± 29.7	0.46	91.2 ± 25.9	88.6 ± 21.0	0.33	79.8 ± 10.7	77.8 ± 11.6	0.47
TR velocity (m/s)	2.3 ± 0.3	2.2 ± 0.3	0.66	2.2 ± 0.3	2.1 ± 0.3	0.26	2.1 ± 0.3	1.9 ± 0.3	0.22
RWT	0.4 ± 0.1	0.4 ± 0.1	0.29	0.4 ± 0.1	0.42 ± 0.07	0.53	0.4 ± 0.1	0.38 ± 0.1	0.51
LV wall thickness (mm)	10.0 ± 1.8	10.0 ± 1.7	0.89	9.7 ± 1.3	9.6 ± 1.3	0.58	9.1 ± 1.1	8.8 ± 1.0	0.27
NT‐proBNP (pg/mL)	643 [239–1738]	215 [137–431]	< 0.001	181 [85–647]	87 [41–165]	< 0.001	56 [40–152]	49 [30–82]	0.35

*Note:* Mean ± SD or median are shown. The abbreviations are shown in Table 1.

Structural and biomarker markers improved in the ≥ 5 and 2–4 groups: LAVI decreased (≥ 5: 49.0 ± 13.7 to 39.3 ± 15.1; 2–4: 38.0 ± 14.9 to 30.4 ± 9.6; both *p* < 0.001) and NT‐proBNP levels declined (≥ 5: 643 [239–1738] to 215 [137–431]; 2–4: 181 [85–647] to 87 [41–165]; both *p* < 0.001). No significant changes occurred in average *E*/*e*′, LVMI, or LV wall thickness.

### 3.4 Factors Associated With Score Improvement

Compared with patients without improvement, those with ≥ 1‐point improvement were older and had more impaired diastolic/structural markers at baseline, including lower septal and lateral *e*′, higher average *E*/*e*′, larger LAVI, and higher NT‐proBNP (Table 3). Rhythm transition pattern (AF → SR, AF → AF, SR → SR) was not associated with score improvement (*p* = 0.63). In multivariable analysis, septal *e*′ and LAVI remained independent predictors of score improvement (adjusted OR: septal *e*′ 0.76 per +1 cm/s [95% CI 0.61–0.92], *p* = 0.004; LAVI 1.07 per +1 mL/m^2^ [95% CI 1.02–1.12], *p* = 0.003) (Table 4).

**TABLE 3:** Comparison of pre‐ablation characteristics between patients with and without improvement in HFA‐PEFF score after catheter ablation.

	Improved (*N* = 89)	Not improved (*N* = 38)	*p*
Clinical characteristic			
Age (years)	67.5 ± 9.5	60.7 ± 14.4	0.002
Sex (male)	62 (69.7%)	30 (79.0%)	0.28
Height (cm)	164.4 ± 9.0	167.5 ± 8.6	0.07
Body weight (kg)	64.6 ± 11.1	66.7 ± 13.6	0.38
BMI (kg/m^2^)	23.8 ± 3.0	23.6 ± 4.0	0.79
Symptomatic	65 (73.0%)	23 (60.5%)	0.16
AF type			
Paroxysmal AF	50 (56.2%)	18 (47.4%)	0.36
Persistent AF	39 (43.8%)	20 (52.6%)	0.36
CHADS_2_ score	1.2 ± 1.1	1.1 ± 0.9	0.77
CHA_2_DS_2_‐VASc score	2.2 ± 1.4	1.9 ± 1.3	0.33
Rhythm transition			
AF to SR (*n* = 48)	34 (38.2%)	14 (36.8%)	0.63
AF to AF (*n* = 2)	2 (2.3%)	0 (0.0%)	
SR to SR (*n* = 77)	53 (59.6%)	24 (63.2%)	
Comorbidities			
Chronic heart failure	13 (14.6%)	9 (23.7%)	0.33
Hypertension	50 (56.2%)	19 (50.0%)	0.66
Diabetes mellitus	9 (10.1%)	6 (15.8%)	0.54
Stroke/TIA	10 (11.2%)	3 (7.9%)	0.80
Antiarrhythmic drug use at baseline			
Class I	19 (21.4%)	10 (26.3%)	0.54
Class II	28 (31.5%)	13 (34.2%)	0.76
Class III	0 (0.0%)	1 (2.6%)	0.12
Class IV	26 (29.2%)	6 (15.8%)	0.11
Medications at baseline			
SGLT2i	7 (7.9%)	4 (10.5%)	0.63
MRA	4 (4.5%)	3 (7.9%)	0.44
ACE‐I or ARB or ARNI	24 (27.0%)	8 (21.1%)	0.48
HFA‐PEFF score			
Septal *e*′ (cm/s)	7.7 ± 2.5	9.2 ± 3.0	0.004
Lateral *e*′ (cm/s)	10.6 ± 3.5	12.2 ± 3.3	0.02
Average *E*/*e*′	9.0 ± 3.6	6.7 ± 2.0	< 0.001
TR velocity (m/s)	2.2 ± 0.3	2.2 ± 0.3	0.71
LAVI (mL/m^2^)	41.7 ± 15.0	31.4 ± 13.5	< 0.001
LVMI (g/m^2^)	91.5 ± 23.1	89.7 ± 31.8	0.73
RWT	0.4 ± 0.1	0.4 ± 0.1	0.30
LV wall thickness (mm)	9.7 ± 1.3	9.5 ± 1.6	0.41
NT‐proBNP (pg/mL)	263 [120–871]	101 [52–381]	0.007

*Note:* Mean ± SD or median are shown. The abbreviations are shown in Table 1.

Abbreviations: AF, atrial fibrillation; SR, sinus rhythm.

**TABLE 4:** Univariable and multivariable logistic regression analyses examining determinants of HFA‐PEFF score improvement.

	Univariable OR (95% CI)	*p*	Multivariable OR (95% CI)	*p*
Clinical characteristic				
Age (+1 year)	0.61 (0.23–1.46)	0.004	0.98 (0.93–1.04)	0.55
Male sex	1.05 (1.02–1.09)	0.29		
Height (+1 cm)	0.96 (0.92–1.00)	0.08		
Body weight (+1 kg)	0.99 (0.95–1.02)	0.38		
BMI (+1 kg/m^2^)	1.02 (0.91–1.14)	0.79		
Symptomatic	1.77 (0.79–3.94)	0.16		
AF type				
Paroxysmal AF	1.42 (0.66–3.07)	0.36		
Persistent AF	0.70 (0.33–1.50)	0.36		
CHADS_2_ score (+1)	1.06 (0.73–1.56)	0.77		
CHA_2_DS_2_‐VASc score (+1)	1.15 (0.87–1.54)	0.33		
Rhythm transition				
AF to SR	1.06 (0.49–2.36)	0.88		
AF to AF	—	0.99		
SR to SR	0.86 (0.39–1.87)	0.70		
Comorbidities				
Chronic heart failure	0.55 (0.21–1.46)	0.22		
Hypertension	1.28 (0.60–2.76)	0.52		
Diabetes mellitus	0.60 (0.20–1.92)	0.37		
Stroke/TIA	2.28 (0.56–15.3)	0.30		
Antiarrhythmic drug use at baseline				
Class I	0.76 (0.32–1.89)	0.54		
Class II	0.88 (0.40–2.01)	0.76		
Class III	—	0.99		
Class IV	2.20 (0.87–6.39)	0.12		
Medications at baseline				
SGLT2i	0.73 (0.21–2.92)	0.63		
MRA	0.55 (0.12–2.90)	0.45		
ACE‐I or ARB or ARNI	1.38 (0.57–3.61)	0.48		
HFA‐PEFF score				
Septal *e*′ (+1 cm/s)	0.82 (0.70–0.94)	0.006	0.76 (0.61–0.92)	0.004
Lateral *e*′ (+1 cm/s)	0.87 (0.77–0.98)	0.02		
Average *E*/*e*′ (+1)	1.42 (1.18–1.77)	< 0.001		
TR velocity (+1 m/s)	1.35 (0.28–6.59)	0.71		
LAVI (+1 mL/m^2^)	1.06 (1.02–1.10)	0.002	1.07 (1.02–1.12)	0.003
LVMI (+1 g/m^2^)	1.00 (0.99–1.02)	0.72		
RWT (+1)	1.29 (0.10–39.45)	0.30		
LV wall thickness (+1 mm)	1.13 (0.86–1.51)	0.41		
log‐transformed NT‐proBNP (+1)	2.91 (1.47–6.11)	0.003	1.54 (0.55–4.42)	0.41

*Note:* The abbreviations are shown in Tables 1 and 3.

## 4 Discussion

### 4.1 Major Findings

This study has three major findings: (1) HFpEF (HFA‐PEFF score ≥ 5) or suspected HFpEF (score 2–4) was highly prevalent, accounting for 83.5% of AF patients undergoing CA (Figure 2); (2) One‐ and two‐year arrhythmia‐free survival rates after CA were similarly high across the three HFA‐PEFF score groups (Figure 3); and (3) CA led to significant reductions in HFA‐PEFF scores, particularly in patients with baseline scores ≥ 5. At one year, all patients with baseline scores ≥ 5 no longer met the HFpEF diagnostic criteria (Table 3), and baseline septal *e*′ and LAVI were independently associated with HFA‐PEFF score improvement after CA in multivariate analysis (Table 4).

### 4.2 Prevalence of HFpEF in AF Ablation Patients

In this study of AF patients undergoing CA, we found a high prevalence of undiagnostic HFpEF as identified by the HFA‐PEFF score. Nearly one‐quarter (23.6%) of patients met diagnostic criteria for HFpEF (score ≥ 5) and an additional 59.8% had intermediate scores suggestive of latent diastolic dysfunction (Figure 2). This underscores that HFpEF is frequently underdiagnosed in the AF population. Our findings are consistent with accumulating evidence that AF and HFpEF frequently coexist. Indeed, large cohort studies report that roughly one‐third of patients presenting with AF meet objective HFpEF criteria even if heart failure was not previously recognized [13]. Similarly, Okada et al. reported that 32.3% of AF patients had an HFA‐PEFF ≥ 5 while only 7.7% carried an HF diagnosis, and the presence of undiagnosed HFpEF was associated with a markedly worse five‐year prognosis [6]. Consistently, our HFpEF patients had a higher risk profile at baseline with older age and more comorbidities which likely contribute to their vulnerability. More recently, a large prospective registry of 1126 AF ablation candidates [14] showed that 47.9% met HFpEF criteria according to at least one contemporary diagnostic algorithm. The high prevalence of undiagnosed HFpEF in AF patients highlights the need for routine assessment of diastolic function and volume status in this population. Application of the HFA‐PEFF scoring algorithm allows for the detection of subclinical HFpEF.

### 4.3 Impact of Baseline HFpEF Severity on Arrhythmia Recurrence

Figure 3 highlights that arrhythmia‐free survival at both 1 and 2 years post‐ablation was virtually identical across the three HFA‐PEFF score groups. This indicates that baseline HFpEF severity did not negatively impact long‐term rhythm outcomes—in fact, patients with more advanced HFpEF features achieved freedom from arrhythmia just as successfully as those with lower HFA‐PEFF scores. A likely explanation is that restoring SR breaks the vicious cycle between AF and diastolic dysfunction, leading to improved filling pressures and reverse atrial remodeling. By maintaining SR, CA may ameliorate HFpEF‐related hemodynamic stress (e.g., reducing left atrial volume and natriuretic peptide levels) so effectively that initial differences in HFpEF severity become less relevant [15, 16].

These findings are in line with the study by Sumiyoshi et al. [17], who also reported no significant differences in AF recurrence rates after CA between patients with baseline high (≥ 5) and low HFA‐PEFF scores. However, their work further demonstrated that AF patients with persistent HFpEF features (HFA‐PEFF ≥ 5) six months after CA had markedly higher three‐year event rates—a composite of cardiovascular hospitalization and all‐cause mortality—compared with those whose scores normalized (27.3% vs. 12.2%), and the post‐ablation HFA‐PEFF score ≥ 5 emerged as a strong independent prognostic indicator [17]. Whether the HFA‐PEFF score at follow‐up can be used to refine prognostic assessment after AF ablation merits further evaluation in larger studies.

### 4.4 Impact of Catheter Ablation on HFA‐PEFF Scores

A principal finding of our study is that successful AF ablation can partially reverse the HFA‐PEFF scores. One year after CA, 70.1% (89/127) of patients demonstrated improvement in the HFA‐PEFF score, and all patients who initially met the diagnostic threshold for HFpEF (score ≥ 5) no longer fulfilled this criterion (Table 3, Figure 4). The structure of score improvement provides mechanistic insight: the reduction in HFA‐PEFF scores was primarily driven by decreases in NT‐proBNP and LAVI, whereas average *E*/*e*′ showed minimal change (Table 2). This pattern strongly suggests that the observed improvement reflects unloading of atrial volume and pressure rather than reversal of intrinsic myocardial diastolic stiffness. This concept is supported by our observation that patients with score improvement had markedly worse baseline LAVI, septal *e*′, and NT‐proBNP (Table 3)—parameters strongly influenced by AF burden. Multivariable analysis identified LAVI and septal e' as independent determinants of score improvement (Table 4), indicating that reverse atrial remodeling represents the dominant mechanism behind HFpEF features resolution. These results align with the findings of Sumiyoshi et al. [17], who demonstrated partial reversion of HFpEF features after CA. However, unlike their cohort—where a subset of patients continued to meet HFpEF criteria despite CA—no patients in our study remained above the HFpEF diagnostic threshold at one year. Whether this reflects differences in population characteristics, rhythm control durability, or disease severity warrants future investigation.

### 4.5 Clinical Implications

The present study offers several clinically relevant insights:

1. HFpEF is highly prevalent yet commonly unrecognized in AF ablation candidates. Incorporating HFA‐PEFF‐based assessment into routine evaluation may improve detection of subclinical HFpEF.
2. CA appears to improve HFpEF‐related cardiac stress in many patients. The findings suggest that when HFpEF‐related abnormalities are largely driven by AF‐associated hemodynamic overload, rhythm control may contribute to their substantial improvement.
3. Baseline HFpEF severity does not predict post‐ablation arrhythmia recurrence. Thus, patients with higher HFA‐PEFF scores should not be considered less likely to benefit from CA on the basis of HFA‐PEFF severity alone.
4. Serial assessment of the HFA‐PEFF score may offer value beyond diagnosis. Normalization post‐ablation could identify patients who have achieved meaningful reversal of AF‐related hemodynamic stress, whereas the clinical implications of persistence of HFpEF features—supported by prior evidence—remain a topic for future investigation.

Overall, these findings support incorporating the HFA‐PEFF score not only for HFpEF detection in AF patients but also as a potential dynamic marker to guide rhythm‐control strategies and long‐term management.

### 4.6 Study Limitations

This study has several limitations. First, it was a single‐center observational study, precluding any causal inference. Second, the relatively small sample size limited our ability to evaluate the association between both baseline and post‐ablation HFA‐PEFF scores and clinical outcomes. Although heart failure events during follow‐up occurred only in patients with high baseline HFA‐PEFF scores (2/30 vs. 0/97), the event number was insufficient to determine whether improvement in the HFA‐PEFF score translated into a reduction in clinical events. Third, AF/AT recurrence may have been underestimated, as detection relied on scheduled follow‐up visits rather than continuous monitoring. Given that several components of the HFA‐PEFF score (e.g., average *E*/*e*′, LAVI) may be influenced by the presence of AF, the observed improvement may reflect, in part, the resolution of AF‐induced hemodynamic stress rather than true HFpEF reversal. Thus, interpretation of HFA‐PEFF dynamics after CA warrants caution. Although symptom‐based criteria are essential for applying the HFA‐PEFF score, this study primarily assessed AF‐related symptoms such as palpitations and exertional dyspnea. Classical heart failure symptoms such as peripheral edema or orthopnea were not systematically evaluated, which may limit the interpretability of the score. Finally, selection bias may have occurred, enriching the cohort with cases of pseudo‐HFpEF predominantly driven by AF. Therefore, the generalizability of our findings to the broader HFpEF population, especially to HFpEF patients without concomitant AF, may be limited.

## 5 Conclusions

HFpEF or suspected HFpEF was common in AF patients undergoing CA, but HFpEF was not linked to mid‐term AF recurrence. CA produced a marked improvement in HFpEF‐related features, and no patients continued to meet the diagnostic criteria for HFpEF one year after the procedure. These findings suggest that, in a substantial proportion of AF patients, HFpEF‐related structural and biochemical abnormalities may be largely driven by AF‐associated hemodynamic stress and can be favorably modified by rhythm control therapy. Beyond arrhythmia suppression, CA may therefore contribute to the reversal of HFpEF‐related pathophysiology in selected patients. The HFA‐PEFF score may facilitate HFpEF screening in AF patients and support the integration of CA into the comprehensive management for patients with HFpEF or suspected HFpEF.

## Author Contributions

M.S. and R.W. wrote the first draft of the protocol manuscript and carry the overall responsibility for the full study and the study protocol. R.W. and Y.O. were substantial contributors to the study concept and design, manuscript drafting, and critical review of the manuscript and will contribute to the acquisition, analysis, and interpretation of the data. M.S., R.W., K.N., Y.S., Y.W., N.O., K.H., S.H., and M.H. contributed to the analysis and interpretation of the data and have approved the final version of this manuscript. Y.O. gave us critical comments on the statistical methods and contributed to the analysis and interpretation of the data.

## Funding

This work is own‐funded.

## Ethics Statement

The study was approved by the Institutional Review Board of Nihon University Itabashi Hospital. Date of Approval: September 25, 2021, Approval number: RK‐210713‐13.

## Consent

The authors have nothing to report.

## Conflicts of Interest

Yasuo Okumura is a professor in an endowed department supported by Boston Scientific Japan, Abbott Medical Japan, Japan Lifeline, Medtronic Japan, and Nihon Kohden. Other authors: No disclosures.

## Data Availability

The authors have nothing to report.
